# Early neurone loss in Alzheimer’s disease: cortical or subcortical?

**DOI:** 10.1186/s40478-015-0187-1

**Published:** 2015-02-10

**Authors:** Thomas Arendt, Martina K Brückner, Markus Morawski, Carsten Jäger, Hermann-Josef Gertz

**Affiliations:** Paul Flechsig Institute of Brain Research, Universität Leipzig, Jahnallee 59, 04109 Leipzig, Germany; Department of Psychiatry, Universität Leipzig, Semmelweisstrasse 10, 4103 Leipzig, Germany

**Keywords:** Nucleus basalis of Meynert, Locus coeruleus, Entorhinal cortex, Neurodegeneration

## Abstract

Alzheimer’s disease (AD) is a degenerative disorder where the distribution of pathology throughout the brain is not random but follows a predictive pattern used for pathological staging. While the involvement of defined functional systems is fairly well established for more advanced stages, the initial sites of degeneration are still ill defined. The prevailing concept suggests an origin within the transentorhinal and entorhinal cortex (EC) from where pathology spreads to other areas. Still, this concept has been challenged recently suggesting a potential origin of degeneration in nonthalamic subcortical nuclei giving rise to cortical innervation such as locus coeruleus (LC) and nucleus basalis of Meynert (NbM). To contribute to the identification of the early site of degeneration, here, we address the question whether cortical or subcortical degeneration occurs more early and develops more quickly during progression of AD. To this end, we stereologically assessed neurone counts in the NbM, LC and EC layer-II in the same AD patients ranging from preclinical stages to severe dementia. In all three areas, neurone loss becomes detectable already at preclinical stages and is clearly manifest at prodromal AD/MCI. At more advanced AD, cell loss is most pronounced in the NbM > LC > layer-II EC. During early AD, however, the extent of cell loss is fairly balanced between all three areas without clear indications for a preference of one area. We can thus not rule out that there is more than one way of spreading from its site of origin or that degeneration even occurs independently at several sites in parallel.

## Introduction

Alzheimer’s disease (AD) is a slowly progressing neurodegenerative disorder associated with a progressive loss of cognitive function eventually leading to dementia. The underlying pathological process is characterized by neuronal cell death, accompanied by typical neuropathological hallmarks, i.e. extracellular deposits of Aß-peptide and intracellular formation of fibrillar aggregates of abnormally phosphorylated tau protein. Depositions of both aggregated Aß and tau are not distributed randomly throughout the brain but rather follow a highly predictive pattern of progression which provides the basis for neuropathological staging of the disease [1,2].

Recent evidence [3] indicates that the pathological process begins years if not decades before clinical symptoms occur. This long “preclinical” phase of AD is of pivotal importance to understand the origin of the disease and may provide a promising time window for potential therapeutic intervention. Exact knowledge on the pathological process occurring during this preclinical phase, however, is difficult to obtain. In a recent systematic survey on more than 2300 nonselected autoptic cases, Braak et al. [4] observed deposits of phospho-tau, immunoreactive for the antibody AT8 already in the first decades of life. Of note, formation of abnormally phosphorylated tau frequently was observed in individuals under 30 years of age and, in addition to the transentorhinal cortex, first became detectable in subcortical nuclei with projection to the cerebral cortex, i.e. locus coeruleus (LC) and nucleus basalis of Meynert (NbM).

It remains unclear at present, however, whether these changes referred to as “pretangles” which represents non-aggregated soluble tau in a hyperphosphorylated form will eventually convert into insoluble PHF-tau. Only a subset of subjects with these changes early in life seem to progress to AD [5]. Even a PHF-like pattern of tau hyperphosphorylation is not necessarily associated with a conversion into aggregated PHF-tau and can occur under certain physiological conditions such as hibernation [6], hypothermia and anesthesia [7] where it is fully reversible. While the pathophysiological significance of so-called “pretangle” tau is not entirely clear, its simultaneous occurrence at both cortical areas and subcortical projection nuclei again brings the old question into new focus of whether degeneration in AD is of subcortical or cortical origin [8-11].

In the mid-late seventies of the 20^th^ century, reductions in the activity of choline acetyltransferase, the biosynthetic enzyme for acetylcholine, [12-14] as well as in the levels of noradrenaline and the activity of its synthetic enzyme dopamine-ß-hydroxylase [15-17] were described in the cerebral cortex of AD patients. As the human cerebral cortex does not contain cholinergic or noradrenergic neurons, these transmitter deficiencies were correctly attributed to a dysfunction in the ascending cholinergic and noradrenergic innervation of the cortex arising in the basal nucleus of Meynert [18,19] and locus coeruleus, respectively. A corresponding loss of neurons in the NbM was first reported in 1982 [20] and rapidly confirmed by others [9,21-24]. Systematic studies clearly indicated that loss of cholinergic NbM neurons occurs early during the course of AD [25,26] and very unlikely is an event secondary to cortical degeneration [10]. The critical role of cholinergic dysfunction in early AD is clearly documented by the fact that pharmacological inhibition of acetylcholinesterase still is the only treatment available for a modest symptomatic therapy during early stages of the disease.

In parallel with descriptions on the cholinergic dysfunction in AD, a neurone loss has consistently been reported for the locus coeruleus [22,27-44]. These cells of the locus coeruleus and nucleus basalis of Meynert share a non-specialised, isodendritic pattern [25,26,45-48] which have led to the suggestion that they may represent a pool of relatively undifferentiated cells with a high susceptibility to degeneration [47,49]. Formation of cortical plaques and tangles and cortical dysfunction where, thus regarded as phenomena secondary to the loss of the ascending inputs [8-11,48]. Still, most systematic surveys on the distribution and progression of neurofibrillary degeneration over the last 25 years or so have focused the attention on the cerebral cortex [1,50-55] where the entorhinal and transentorhinal cortex appear to be affected most early. Accordingly, a pathogenetic concept has been formulated assuming the process of degeneration starts in the transentorhinal cortex from where it spreads throughout the brain [56]. Still, more recent findings on early subcortical “pretangle tau pathology” in nonthalamic subcortical nuclei innervating the cortex, including LC and NbM, have again challenged this concept and a potential subcortical origin of neurodegeneration was again acknowledged [56].

Until now, only a few studies with a restricted number of patients have compared neuronal loss as a direct quantitative measure of degeneration in the NbM and LC, and to the best of our knowledge there is no study comparing cell loss in the ascending projection neurons of the NbM and LC with those in the entorhinal cortex. Therefore, to contribute to the identification of the early site of degeneration in AD, here, we address the question whether cortical or subcortical degeneration occurs more early and develops more quickly during progression of the disease. We have assessed by stereological methods neurone counts in the NbM, LC and entorhinal cortex of the same individual autopsy cases covering a wide spectrum of disease stages from preclinical AD to severe dementia.

## Materials and methods

### Patients and healthy controls

Brain tissue of 101 AD patients and 19 healthy controls dying without any history of neurological or psychiatric illness was used. The diagnosis of AD was made on the basis of both clinical and neuropathological evidence according to the criteria of the International Working Group (IWG) for New Research Criteria for the diagnosis of AD [57,58] in the revision of 2014 (IWG-2) [59], the NIA-AA diagnostic criteria in the revision of 2011 [3,60-62] and the NIA-AA guidelines for the neuropathological assessment of AD [63,64]. Only cases with typical AD according to IWG-2 criteria were included. Cases with history of stroke as well as other central nervous system disorders such as tumors, inflammation, Lewy body disease or frontotemporal dementia and premortem hypoxia related to agonal states were excluded from the present study. Cases with substantial microvascular pathology such as cortical microinfarcts, deep white matter and periventricular demyelination, silent lacunar infarcts and extensive leukoaraiosis as well as cases with argyrophilic grain disease were also excluded. All cases underwent neuropsychological assessment within the last 6 months prior to their death. Clinical Dementia Rating (CDR) scale scoring was based on neuropsychological testing (CERAD) [65], MMSE [66] and rating scales [67]. CDR scale score was used to assign cognitive function to five levels defined as no memory loss (CDR 0), questionable dementia (CDR 0.5), mild dementia (CDR 1), moderate dementia (CDR 2), and severe dementia (CDR 3) [68]. All cases were neuropathologically assessed for NFT stage according to Braak and Braak [1], and Braak et al. [50], for Aß/amyloid plaque score according to Thal et al. [2] and for neuritic plaque score according to CERAD [69]. NFTs and Aß/amyloid plaques were detected by immunocytochemical labeling of phospho-tau (anti-human PHF-tau monoclonal antibody AT8; Thermo Scientific) and Aß (beta amyloid monoclonal antibody, 6E10; BioLegend), respectively. Severity of AD pathology was scored following the consensus guidelines for the neuropathologic evaluation of Alzheimer’s Disease according to Hyman et al. [63] and Montine et al. [64]. Case recruitment, autopsy and data handling have been performed in accordance with the ethical standards as laid down in the 1964 Declaration of Helsinki and its later amendments as well as with the convention of the Council of Europe on Human Rights and Biomedicine and had been approved by the responsible Ethics Committee of Leipzig University.

Based on neuropsychological assessment (CDR) and neuropathological examination [63,64], cases were allocated to one of the following six groups.

- 1. Controls. Individuals with a complete absence of clinical symptoms of AD (CDR = 0) and of AD pathology (ABC-score=“Not”) were considered as controls.
- 2. Preclinical AD. Individuals with a complete absence of clinical symptoms of AD (CDR = 0) and the presence of initial AD pathology, scored as ABC-score=“Low” were considered as preclinical cases of AD (asymptomatic at risk according to IWG-2; preclinical AD stages 1 and 2 according to NIA-AA).
- 3. Prodromal AD/MCI. Individuals with early cognitive symptoms not reaching severity of dementia (CDR = 0.5) in combination with the presence of AD pathology, reaching an ABC-score of “Low” or “Intermediate” were considered as prodromal AD according to IWG-2 and AD-MCI according to NIA-AA criteria.
- 4–6. AD dementia. Individuals with some form of dementia (CDR between 1 and 3) in combination with a ABC-score of “Intermediate” or “High” and the absence of exclusion criteria (see above) were considered typical AD dementia according to IWG-2 and and NIA-AA criteria and indexed according to their CDR-score as mild AD (Group 4), moderate AD (Group 5) and severe AD (Group 6).

### Tissue processing and stereological analysis

Brains were immersed in 4% formaldehyde in phosphate buffer (0.1 M; pH 7.4) for one month. Tissue blocks containing the entorhinal cortex, basal nucleus of Meynert complex and the locus coeruleus were immersed in 30% sucrose for cryoprotection and cut in the coronal plane on a freezing microtome at a microtome setting of 30 μm. Every 10th section was collected for sampling.

Total neurone number in the NbM, LC and Layer II of the entorhinal cortex was determined by the unbiased stereological method of the optical fractionator [70]. Cholinergic neurons of the nucleus basalis Meynert complex, consisting of the medial septal nucleus (Ch1), nucleus of the vertical limb of the diagonal band (Ch2) and substantia innominate (Ch4) were identified by immunocytochemical detection of choline acetyltransferase [26,71] using a goat anti-choline acetyltransferase polyclonal antibody (AB 144P Chemicon) as described [72]. Boundaries of the NbM complex were identified based on a previous three-dimensional reconstruction comprising the subpopulations of Ch1, Ch2, Ch4am, Ch4al, Ch4i and Ch4p [9,73]. Neurons were sampled over the entire length of the NbM complex extending from the septum (Ch1) to its most posterior parts (Ch4p) at the premammillary level posterior to the ansa peduncularis. Neurons of the locus coeruleus were identified by the presence of neuromelanin [74,75]. In the present study, the term locus coeruleus (LC) refers to both the nucleus coeruleus and nucleus subcoeruleus. For outlining layer II of the entorhinal cortex, we followed the criteria of Amaral and Insausti [76] and Insausti et al. [77]. The entorhinal cortex was sampled throughout its entire length extending from 2 to 3 mm behind the frontotemporal junction (limen insulae) to the rostral pole of the lateral geniculate nucleus.

Neuronal counts were performed on Nissl-counterstained sections on a Zeiss Axiophot, equipped with a motorized stage (Märzhäuser, Wetzlar, Germany), a Ludl MAC 5000 (LEP, Hawthorne, NY, USA) and a digital camera CX 9000 (MicroBrightField, Williston, VT, USA). The anatomical boundaries of the considered region were outlined using a 5x lens and the reference volume for each structure was determined from these areas encircled on each section averaged over the entire length of the structure (principle of Cavalieri). The disector was 150x150μm wide and 10 μm in height. Post-processing shrinkage of tissue resulted in a final section thickness of 18 +/− 2 μm, which permitted a consistent sampling of 10 μm with the dissector and the use of guard zones of at least 3 μm on either side of the section. On average, 300 – 600 profiles were counted with a 20x lens in each case and region.

The intra individual coefficient of error (CE) for the individual estimates of cell numbers ranged from 0.01 to 0.08 with an overall average of 0.046. The inter-individual coefficient of variation (CV) within each of the six different groups ranged from 0.0081 to 0.372. The ratio CE^2^/CV^2^, thus, ranged from 0.005 to 0.25, indicating that the precision obtained with the sampling scheme was sufficient for optimal sampling [70].

To make the changes in neurone number comparable obtained for the three different regions at different stages of AD, we calculated the effect size “*d”* of cell loss compared to the appropriate control group according to Hedges and Olkin [78]. Effect size “*d*” is defined as the mean of controls (indexed as “_1_”) minus the mean of AD (indexed as “_2_”), divided by the pooled standard deviation “s*” (n: sample size; s: standard deviation).

$$ d=\frac{{\overline{x}}_1-{\overline{x}}_2}{s*}\cdot \kern1em s*=\sqrt{\frac{\left({n}_1-1\right){s}_1^2+\left({n}_2-1\right)s}{n_1+{n}_2-2}} $$

## Results

### Both cortical and subcortical cell loss can be detected already at prodromal stages of Alzheimer’s disease

**Figure 1:**
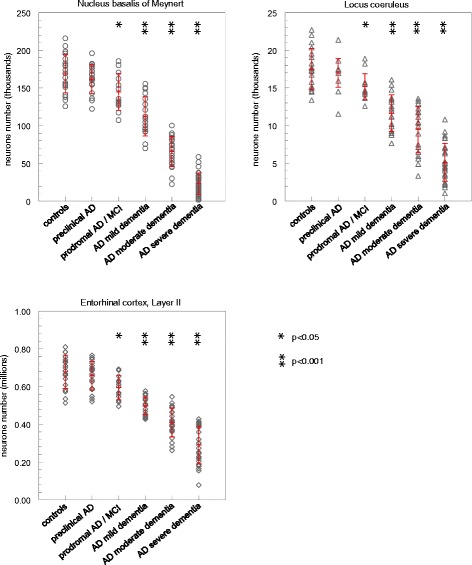
**Total number of neurons in the nucleus basalis of Meynert, locus coeruleus and entorhinal cortex layer II (data are individual values for each brain together with mean values [+/−SD] for each group [red]; Student’s t-test for comparison to controls).**

In the normal aged brain (mean age 81.3 +/− 4.4 years; see Table 1), we determined the mean number of neurons in the NbM, in the LC and in Layer II of the entorhinal cortex at 168,834+/−25,573, 17,487+/−2,736 and 677,685+/−88,072, respectively. As can be seen in Figure 1, there was, however, a rather large inter-individual variety and neuron count varied from 125,734 to 215,827 in the NbM (CV = 0.15), from 13,371 to 22,712 in the LC (CV = 0.15) and from 512,914 to 810,211 in the entorhinal cortex, layer II (CV = 0.13).

**Table 1:** **Synopsis of cases**

	**Control**	**Alzheimer’s disease**				
		**Preclinical AD**	**Prodromal AD/MCI**	**AD mild dementia**	**AD moderate dementia**	**AD severe dementia**
**Number of cases**	19	18	13	18	22	30
**Gender: male/female**	8/11	7/11	6/7	8/10	9/13	12/18
**Age in years (+/-SD)**	81.3 +/- 4.4	79.9 +/- 6.5	80.2 +/- 5.4	81.4 +/- 5.9	83.6 +/- 5.8	83.4 +/- 6.8
**CDR**						
0	19	18				
0.5			13			
1				18		
2					22	
3						30
NFT stage*						
0	19					
1		18	6			
2			7	6	4	
3				12	18	30
Aß/amyloid plaque score*						
0	19					
1		18	5			
2			8	4	2	
3				14	20	30
CERAD*						
0	19					
1		18	4			
2			9	5	2	
3				13	20	30
ABC score*						
Not	19					
Low		18	7			
Intermediate			6	6	4	
High				12	18	30
**Cause of death**						
Bronchopneumonia	5	15	10	12	16	25
Myocardial infarction	8					
Congestive heart failure			1	1	1	2
Embolism of lung	3	3	1	2	1	2
Sepsis	1			2	3	1
Lung cancer	1			1		
Pancreatic cancer	1					
Breast cancer					1	
Renal failure			1			
**Postmortem delay in h (+/-SD)**	3.4 +/- 0.6	3.8 +/- 0.7	4.1 +/- 0.5	3.5 +/- 0.6	3.7 +/- 0.7	3.6 +/- 0.8
**Fresh brain weight in gm (+/-SD)**	1278 +/- 76	1258 +/- 51	1245 +/- 86	1212 +/- 56	1183 +/- 64	1150 +/- 103
**Phospho-tau (mab AT8) positive pathology**						
Ncl basalis of Meynert	0	18	13	18	22	30
Locus coeruleus	0	18	13	18	22	30
Entorhinal cort. Layer II	0	18	13	18	22	30
**Aß (mab 6E10) positive pathology**						
Ncl basalis of Meynert	0	0	8	18	22	30
Locus coeruleus	0	0	0	14	20	30
Entorhinal cort. Layer II	0	6	10	18	22	30

*according to [64].

In AD, there occurred a progressive loss of neurons in all three areas which paralleled the progression of the disease. Also, there was a large individual range in neurone count for each group. At preclinical stages of AD, neurone count in all three areas tended to be reduced. Cell loss, however, was insignificantly small (NbM: 4.3+/−2.6%; LC: 3.0+/−2.5%; entorhinal cortex, layer II: 3.1+/−2.6%). Still, at stages of prodromal AD/MCI, neurone loss was significant at a level of p < 0,05 in all three areas, amounting to 14.36+/−4.0% in the NBM, 13.33+/−2.7% in the LC and 12.45+/−2.6% in the entorhinal cortex, layer II. AT8 immunoreactive PHF-tau could be detected in all three areas already at preclinical as well as at all subsequent stages (Table 1).

Neurone loss further progressed in the groups of mild dementia (NbM: 33.48+/−3.6%; LC: 33.5+/−3.3%, entorhinal cortex, layer II: 26.6 + 7-1.6%) and moderate dementia (NbM: 61.0+/−2.5, LC: 46.0+/−3.7%, entorhinal cortex, layer II: 39.6+/−2.4%). At the most advanced stages (AD severe dementia), average neurone loss reached 83.3+/−1.53% in the NbM, 70.8+/−2.6% in the LC and 57.2+/−2.6% in the entorhinal cortex, layer II.

### Effect size of cell loss is largest in the basal nucleus, in particular at more advanced stages of the disease

**Figure 2:**
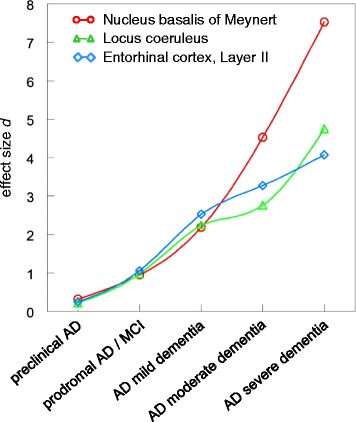
**Comparison of effect size** ***d*** **for neurone loss in the nucleus basalis of Meynert, locus coeruleus and entorhinal cortex layer II.**

To make the effects of AD on neurone number comparable between the different regions, we calculated the effect size “*d”* of cell loss compared to the appropriate control group (Figure 2). At preclinical AD, effect size matched the criteria for a “small effect” (defined as 0.2 < *d* < 0.3) [79,80]. It was somewhat larger in the NbM (NbM: *d* = 0.32), compared to LC and entorhinal cortex, layer II (LC: *d* = 0.22; entorhinal cortex, layer II: *d* = 0.25). At prodromal AD/MCI, effect size matched the criteria of a “large effect” (defined as *d* > 0.8) [79,80]. At this stage of the disease, it was basically indistinguishable between the three areas (NbM: *d* = 0.95; LC: *d* = 0.98; entorhinal cortex, layer II: *d* = 1.06).

For the more advanced stages of moderate AD and severe AD, effect size was clearly highest in the NbM (*d* = 4.53 and *d* = 7.53), while its increase was somewhat less pronounced for both the LC (*d* = 2.67 and *d* = 4.75) and entorhinal cortex, layer II (*d* = 3.28 and *d* = 4.01) (Figure 2).

### Both cortical and subcortical neurone loss show a nearly simultaneous onset

**Figure 3:**
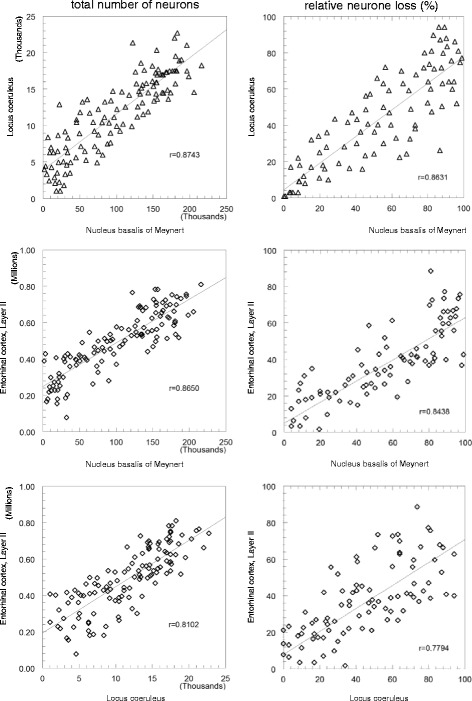
**Comparison of cell loss in the three different areas.** Both total neurone number and relative neurone loss in each of the regions were plotted against those in the other two. All relationships were significant at p < 0.001; r: coefficient of correlation according to Bravais-Pearson.

For a more direct comparison of cell loss in the three different areas, both absolute neurone number and relative neurone loss in each of the regions were plotted against those in the other two (Figure 3). Overall, there was a very high degree of correlation of neurone loss in each of the three areas with those in the other two areas (correlation coefficient r ranging from 0.7794 to 0.8631; p < 0.001).

Further, the intersections of the regression lines of cell loss between different areas (right panel of Figure 3) are all close to 0, indicating a nearly simultaneous onset of cell loss in those areas.

## Discussion

Most studies agree on the fact that both NbM and LC show the greatest cell loss among subcortical areas in AD. Still, only a few original reports, reviews or meta analyses have directly compared the extent of cell loss in the NbM and LC. To the best of our knowledge none of them has compared neurone loss in these subcortical areas to those in the entorhinal cortex within the same subjects analyzing different stages of the disease.

Here, we report on neurone number in these brain areas obtained by stereological techniques on 101 AD patients and 19 healthy controls. Based on neuropsychological assessment and neuropathological examination, AD patients were allocated to one of 5 stages reflecting increasing disease progression.

### Neurone counts in healthy controls

For the cohort of healthy controls, we determined neurone numbers in the NbM, LC and entorhinal cortex layer II which are in very good agreement with previous studies. For the NbM, we determined a neurone number of 125,734 to 215,827, with a mean of 168,834+/−25,573. Previous studies on normal elderly of about 80 years of age reported a mean neurone count in the NbM of 167,200 to 178,400 with individual cell counts ranging from 133,800 to 296,000 [9,26,73].

For the LC, we determined a neurone number of 13,371 to 22,712, with a mean of 17,487+/−2,736. This is very close to previous estimates by unbiased stereological techniques where cell counts varied between 15,731 and 18,307 [30,81]. For comparison, previous studies applying various different stereological and non-stereological counting methods to the LC reported on mean cell numbers between 11,500 and 19,100 with individual cell counts ranging from about 6,000-11,000 to about 27,000-30,000 [28,30,33,37,81-86].

For the entorhinal cortex, layer II neurons, we determined numbers of 512,914 to 810,211 with mean values of 677,685+/−88,072. For comparison, previous stereological studies obtained mean numbers between 607,200 and 779,000 [87-90].

### The range of percentage estimates of cell loss in Alzheimer’s disease is large

Previous studies on the NbM, LC [22,28,31,33,35,37,41,42,44,84,91] and entorhinal cortex layer II [87-90,92] show that the extent of individual cell loss in AD varies to a great extent with reported figures of a few percent up to 90% cell loss or even more. To some extent, this variability can be explained by differences in the age and disease stage of the patients which not always has been controlled for as well as by different sampling and counting protocols [90]. Still, even within each of the 5 stages of AD defined by neuropsychological and neuropathological criteria we have analyzed here separately, cell number shows a rather wide variability. Also, a meta-analysis [93] reported on similar magnitudes in effect size when comparing stereological to non-stereological studies on NbM and LC, suggesting that the observed differences might reflect true biological variability. In this meta-analysis, effect size ranged from 0.41 to 7.28 in the NbM and from 1.12 to 4.24 in the LC [93]. In the present study, we obtained a comparable effect size between 0.95 and 7.53 for the NbM and between 0.98 and 4.75 for the LC, at stages of prodromal AD and at AD with severe dementia, respectively.

In all three brain areas analyzed, neurone loss, still insignificantly small by statistical means, became detectable already at preclinical AD. The simultaneous presence of AT8 positive NFTs strongly suggests a pathogenetic link towards fibrillary tau pathology. It must remain open, however, at present to what extent these degenerative changes represent localized disease-specific pathology or might be attributed to an accelerated aging process [94].

### Comparing cortical versus subcortical cell loss

A few studies described a somewhat greater neurone loss in the NbM compared to LC. Jellinger et al. [95] reported on a somewhat more severe involvement of the NbM (15–90% neuron loss), compared to LC (4–88% cell loss). Similarly, Moll et al. [40] described a much larger neurone loss in the NbM than in the LC. In a comprehensive meta-analysis, Lyness et al. [93] reported on a somewhat larger effect size of neurone loss in the NbM (effect size ranged from 0.41 to 7.28; mean d = 2.48, median d = 3.03; 33 studies) compared to the LC (effect size ranged from 1.12 to 4.24; mean d = 2.28; median d = 2.62; 24 studies).

On the contrary, Geula and Mesulam [96] reported on a 20–70% cell loss in the Ch4 area of the NbM compared to a 40–80% cell loss in the LC. Mann et al. [22,97], Ichimiya et al. [36] and Wilcock et al. [43] also described a somewhat greater loss of neurons in the LC than in the NbM, in particular in patients under 80 years of age. Also, Zarow et al. [98] reported on a more pronounced neurone loss in the LC compared to NbM.

Förstl et al. [99] clearly showed a relationship between the history of depression and depletion of noradrenergic neurons in the LC. In a prospective study, they observed in a subgroup of AD patients with a history of depression a significantly lower neurone number in the LC and slightly higher neuronal density in the NbM. Chan-Paly and Asan [31] also described a loss (−55%) of LC neurons in chronically depressed patients without dementia. Still, Syed et al. [100] and Hoogendijk et al. [82] failed to observe a link between depression and cell loss in the LC in AD.

Our study clearly shows that at more advanced stages of AD, i.e. AD with moderate or severe dementia, cell loss is most pronounced in the NbM, while the layer II entorhinal cortex neurons are least affected and the LC shows an intermediate extent of cell loss. This is basically in agreement with the meta-analysis on NbM and LC by Lyness et al. [93] where maximal effect sizes were clearly higher for the NbM than for LC.

Still, the situation is much less conclusive for the very early, i.e. preclinical stages of AD. The question where the pathological process in the brain originates is still controversial. Early studies in the eighties of the last century had documented a contribution towards cortical pathology of ascending cholinergic innervation arising in the basal forebrain, suggesting a subcortical origin of AD pathology [8,9,11]. Still, the currently prevailing concept postulates an origin within the transentorhinal cortex from where pathology spreads throughout the cortex [1]. Recent findings on “pre-tangle” tau pathology in subcortical projection nuclei, including LC and NbM in individuals under 30 years of age [4,56], however, have challenged this concept. Consequently, Braak et al. [4] recently revised the NFT stages and included “pre-tangle stages” in nonthalamic subcortical nuclei giving rise to cortical innervation. In his survey on more than 2300 nonselected autopsy cases between 1 and 100 years of age, he observed in a few cases a widely distributed subcortical tau pathology, predominantly in the LC, in the absence of cortical tau pathology. He, thus concluded on an origin of tauopathy associated with sporadic AD in the lower brainstem nuclei rather than in the transentorhinal region [4,101]. Still, there are arguments which call into question an origin of pathology in the LC. While the severity of tau pathology in the LC increases with increasing NFT stages, there are still cases with considerable entorhinal tau pathology and only minimal amounts of tau pathology seen in the LC: It has, thus, been argued [102] that the LC becomes increasingly involved during AD progression rather than being the site initially affected.

Our findings show that both entorhinal cortex layer II neurons and subcortical neurons in the NbM and LC are reduced in number already at stages of prodromal AD/MCI. First hints of cell loss, albeit still insignificantly small, even occur already at preclinical stages, i.e. in the complete absence of clinical symptoms. While throughout all stages of the disease, some cases tend to show a more severe involvement in one area than the other (Figure 3, right panel), the overall pattern of cell loss appeared to be fairly balanced between all three areas. We thus failed to observe a systematic pattern which would allow to conclude on an early site of lesion and a subsequent progression of pathology from one area to another.

To identify an initial site of degeneration if it exists, it might be necessary to go even further back on the preclinical time scale. Then, however, we run into the problem of how to recognize prospective AD subjects. It might, thus, be too early to draw any conclusion on whether AD pathology is of cortical or subcortical origin. Also, we need to consider that there might be more than one way of speading of pathology from its site of origin giving rise to different neuropathologically defined subtypes of AD [103]. We even can not rule out at present that degeneration occurs independently at several sites in parallel.

### Ethical approval

All procedures performed in studies involving human participants were in accordance with the ethical standards of the institutional and/or national research committee and with the 1964 Helsinki declaration and its later amendments or comparable ethical standards.
